# Subtle Differences to Make the Difference

**DOI:** 10.1097/HS9.0000000000000038

**Published:** 2018-04-05

**Authors:** Melania Tesio

**Affiliations:** Institut Necker Enfants Malades, Paris, France

In a recent Nature Medicine issue, 2 groups developed novel chimeric antigen receptor (CAR) T cell-mediated therapies against multiple myeloma and T-cell malignancies.

Genetically engineered T cells have opened a new era in onco-hematology, as recently reviewed.^1^ Paradigmatic examples of this revolution are chimeric antigen receptor (CAR) T cells, autologous T lymphocytes engineered ex vivo to express CAR. These artificial receptors are fusion proteins that incorporate an antibody-derived antigen-recognition domain, a transmembrane region, intracellular costimulatory domains and T-cell receptor (TCR) activation domains. Thanks to this molecular structure, CAR T lymphocytes are able to physically engage with cancer-associated cell surface antigens and to elicit cytotoxic signaling in tumor cells, leading to their death.

To date, CAR T cells targeting CD19 have been the most successful ones, inducing complete and durable remissions in patients with refractory and relapsed B-cell malignancies such as acute lymphoblastic leukemia.^2^ These unprecedented results rely, at least in part, on the uniform expression of CD19 on malignant B cells. The scenario is different in other malignancies, such as multiple myeloma (MM), characterized by intrapatient heterogeneity in surface antigen expression.^3^ In B-cell malignancies, moreover, the therapeutic effects of CAR T cells have not been undermined by the side effects linked to the lack of CAR target selectivity. The depletion of normal B cells, which also express CD19, can be handled clinically through antibody injections. Antigen selectivity is, however, extremely important for CAR T cell-mediated therapies of other hematological tumors, such as T-cell malignancies, since depletion of healthy T lymphocytes would lead to severe immunosuppression. The lack of antigen selectivity and/or its uniform expression on cancer cells have thus hampered the development of immunotherapies against MM and T-cell malignancies. In a recent issue of *Nature Medicine*, 2 groups reported novel strategies to overcome these limitations.

**Figure 1:**
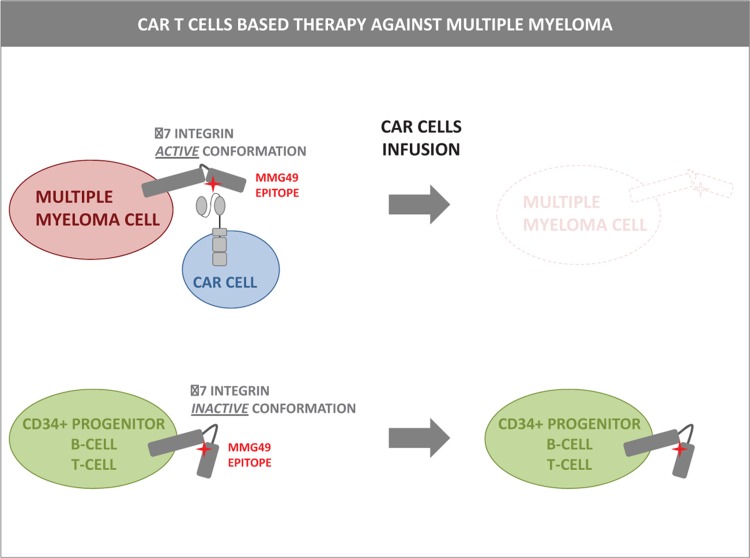
**CAR T cells target the active conformation of β7 integrin on multiple myeloma cells.**

By screening more than 10,000 anti-MM hybridomas, Naoki Hosen and colleagues identified MMG49, a monocolonal antibody that exhibited extensive binding in 45/51 MM samples but minor recognition of normal leukocytes and non-MM cells.^4^ Further characterization of this antibody revealed that MMG49 recognizes a conformation-sensitive epitope on the β7 integrin. This consists of 12 amino acid residues that become exposed when, in response to activation signals, the β7 integrin transits from a closed, low-ligand affinity state to an open, and high-ligand affinity conformation. Interestingly, whereas β7 integrin was expressed at low or moderate levels on B cells, T lymphocytes, and CD34+ hematopoietic progenitor cells, its activated conformation was detected most specifically on MM cells. When engineered to recognize this conformation-active epitope, CAR T cells exhibited pronounced in vitro cytotoxic activity against primary MM cells but not against CD34+ progenitors, peripheral blood mononuclear cells, or activated T cells. In xenograft models, moreover, MMG49 CAR T cells significantly decreased tumor burden. Conversely, they did not kill engrafted human CD34+ progenitors or B cells, thus demonstrating that cancer-associated conformational changes represent selective targets for immunotherapy against MM (Fig. 1).

**Figure 2:**
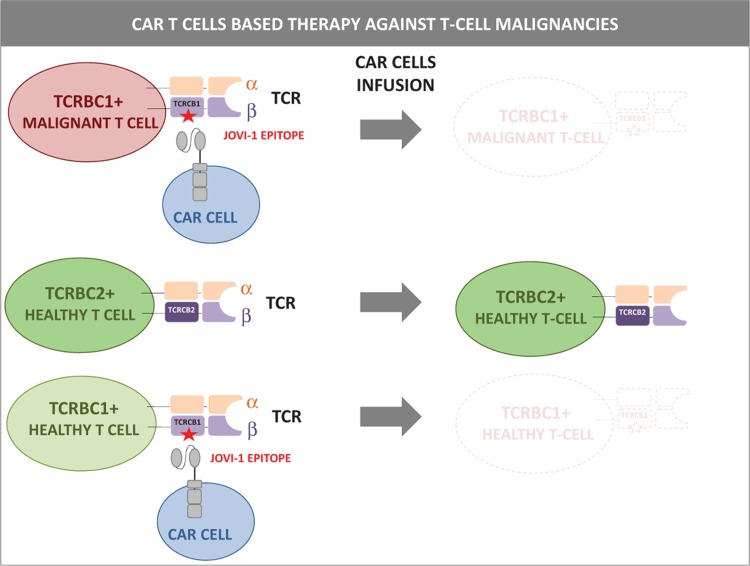
**CAR T cells selectively recognizing TCRBC1 + malignant mature T cells target tumor cells while partially preserving the T-cell repertoire.**

Differences in the expression of the TCR β chain were exploited by Paul Maciocia and colleagues to develop CAR T cells selectively targeting T-cell tumors.^5^ Given its uniform expression on most mature malignant T cells but also on the majority of their healthy counterparts, the TCR αβ is an interesting but highly unselective therapeutic antigen. Maciocia and colleagues exploited the fact that the TCR β chain component of the TCR αβ heterodimer is constituted by a C-terminal constant region encoded by either the *TCRBC1* or the *TCRBC2* gene. Since TCRBC1 and TCRBC2 are expressed in a mutually exclusive manner, the healthy population of polyclonal T lymphocytes comprises a mixture of TCRBC1+ and TCRB2+ cells. Being clonal, malignant T cells, in contrast, express either TCRBC1 or TCRBC2. Hence, targeting TCRBC1 in TCRBC1+ malignancies (or TCRBC2 in TCRBC2+ tumors) is expected to produce therapeutic benefits without depleting the entire healthy T-cell population (Fig. 2). To prove this concept, Maciocia and colleagues developed JOVI-1, a monoclonal antibody that targets TCRBC1+ cells in a selective manner by recognizing 2 amino acid residues specifically exposed in TCRBC1 but not in TCRBC2. When armed against the JOVI-1 epitope, CAR T cells killed primary malignant T cells derived from different lymphoproliferative disorders (ie, T-cell prolymphocytic leukemia, peripheral T-cell lymphoma not otherwise specified and adult T-cell leukemia/lymphoma) but they spared TCRBC2+ cells. Once tested in vivo, TCRBC1 CAR T cells depleted T-cell acute lymphoblastic leukemia cells in xenograft models of the disease. Importantly, although CAR T cells depleted healthy TCRBC1 + T lymphocytes (comprising approximately 35% of the peripheral blood T-cell population), this is not expected to lead to a complete loss of cellular immunity. As suggested by in vitro experiments, the T-cell response against viral infections was not skewed toward TCRBC1 or TCRBC2-expressing cells but involved both subpopulations of T cells.

Although both Mociocia and Hosen presented preclinical evidence relying exclusively on cell line-derived xenograft models, their studies are innovative and they demonstrate that subtle differences in tumor-associated antigens could be exploited to develop selective CAR T cells. A few questions, however, remain open. Despite the fact that MM cells did not undergo antigen escape, a subset of mice receiving MMG49 CAR T cells relapsed. More work will thus be necessary to understand the reasons behind this phenomenon and to evaluate to which extent the active conformation of β7 integrin is superior to CD269, a therapeutic antigen showing the most promising results in early clinical trials of MM-targeting CAR T cells.^6^ Moreover, although TCRBC1 + CAR T cells did not drive total loss of cellular immunity, side effects may still result from reducing the T-cell repertoire to only TCRBC2 + or TCRBC1+ cells. Clarifying these aspects will be important to further translate these interesting studies into the clinical setting.
